# Characterizing microclimate in urban malaria transmission settings: a case study from Chennai, India

**DOI:** 10.1186/1475-2875-12-84

**Published:** 2013-03-02

**Authors:** Lauren J Cator, Shalu Thomas, Krijn P Paaijmans, Sangamithra Ravishankaran, Johnson A Justin, Manu T Mathai, Andrew F Read, Matthew B Thomas, Alex Eapen

**Affiliations:** 1 Department of Entomology, Center for Infectious Disease Dynamics and Department of Entomology, Merkle Lab, The Pennsylvania State University, University Park, 16802, PA, USA; 2 National Institute of Malaria Research (ICMR), IDVC Field Unit, NIE Campus, 2nd Main Road, TNHB, Ayapakkam, 600 077, Chennai, India; 3 Department of Zoology, Madras Christian College, Tambaram, 600 059, Chennai, India; 4 Center for Infectious Disease Dynamics, The Pennsylvania State University, University Park, 16802, PA, USA

**Keywords:** Temperature, Extrinsic incubation period, *Anopheles stephensi*, Urban malaria

## Abstract

**Background:**

Environmental temperature is an important driver of malaria transmission dynamics. Both the parasite and vector are sensitive to mean ambient temperatures and daily temperature variation. To understand transmission ecology, therefore, it is important to determine the range of microclimatic temperatures experienced by malaria vectors in the field.

**Methods:**

A pilot study was conducted in the Indian city of Chennai to determine the temperature variation in urban microclimates and characterize the thermal ecology of the local transmission setting. Temperatures were measured in a range of probable indoor and outdoor resting habitats of *Anopheles stephensi* in two urban slum malaria sites. Mean temperatures and daily temperature fluctuations in local transmission sites were compared with standard temperature measures from the local weather station. The biological implications of the different temperatures were explored using temperature-dependent parasite development models to provide estimates of the extrinsic incubation period (EIP) of *Plasmodium vivax* and *Plasmodium falciparum*.

**Results:**

Mean daily temperatures within the urban transmission sites were generally warmer than those recorded at the local weather station. The main reason was that night-time temperatures were higher (and hence diurnal temperature ranges smaller) in the urban settings. Mean temperatures and temperature variation also differed between specific resting sites within the transmission environments. Most differences were of the order of 1-3°C but were sufficient to lead to important variation in predicted EIPs and hence, variation in estimates of transmission intensity.

**Conclusions:**

Standard estimates of environmental temperature derived from local weather stations do not necessarily provide realistic measures of temperatures within actual transmission environments. Even the small differences in mean temperatures or diurnal temperature ranges reported in this study can lead to large variations in key mosquito and/or parasite life history traits that determine transmission intensity. Greater effort should be directed at quantifying adult mosquito resting behaviour and determining the temperatures actually experienced by mosquitoes and parasites in local transmission environments. In the absence of such highly resolved data, the approach used in the current study provides a framework for improved thermal characterization of transmission settings.

## Background

Temperature is one of the key environmental factors influencing the dynamics and distribution of malaria. Various studies show that mosquito population dynamics [1-3], frequency of blood feeding [4], parasite fitness in the vector [5], mosquito immune processes [6] and the extrinsic incubation period (EIP) of the parasite within the mosquito [7,8] are all affected by temperature.

Currently, the majority of studies considering the effect of temperature on mosquito bionomics and malaria risk use temperatures recorded from standard outdoor weather stations [9-16]. However, temperatures from weather stations, which are often separated from transmission sites, do not necessarily represent the temperatures experienced by vectors in local transmission settings in the field [17]. Temperature can vary greatly between indoor and outdoor environments and also be influenced strongly by local features such as house design, house materials, and vegetation cover [3,17-20]. Such differences can translate to marked variation in mosquito life history and estimates of malaria transmission [17].

Further, most studies characterize the environmental conditions using measures of temperature such as mean monthly temperatures, yet recent theoretical [21] and empirical [22] work has demonstrated that daily fluctuations in temperature also affect mosquito and parasite traits that determine transmission processes. In order to understand the influence of temperature on transmission, temperature needs to be measured within the actual environment inhabited by mosquito vectors and at a scale relevant to mosquito and parasite biology. The current paper presents the results of a pilot study, providing a basic methodological approach for addressing these issues.

The study was conducted in the city of Chennai in Tamil Nadu, southeastern India, where the predominant malaria vector is *Anopheles stephensi*. This species is an important vector of malaria throughout the Indian subcontinent and has been reported to rest both indoors [23-26] and outdoors [23]. Data from mechanical aspiration and pyrethrum spray catches, in which a home is sealed and pyrethrum fog is applied to capture all knocked-down mosquitoes on white sheets [27], in India have documented this species resting in a variety of indoor structures [23-25], as well as in semi-outdoor habitats such as cattle sheds [25]. The majority of *An. stephensi* females were found to leave indoor resting habitats early in the evening (18.30 hrs.), feed primarily outdoors, and then return to indoor resting habitats in the middle of the night (23.30 hrs.) [23]. However, there seems to be plasticity in peak biting times with one study reporting two peaks of activity [28].

The biting and resting behaviour of *An. stephensi* within urban slum transmission sites in Chennai is not well characterized, although adults have been sampled periodically from a range of indoor and outdoor structures. In the absence of more explicit knowledge, the current study describes methods for recording temperature in a range of potential indoor and outdoor resting locations in order to capture the broad ‘thermal envelope’ available within the local transmission settings. These diverse temperature data were then used to drive thermodynamic models of parasite development to examine the expected variation in the Extrinsic Incubation Period (EIP) of malaria parasites across the transmission environment. The logic is that in the absence of precise knowledge of mosquito resting behaviour (and given that mosquitoes almost certainly distribute across a range of microhabitats during their adult life) the transmission environment is better characterized as an ensemble mean of available habitats with an upper and lower bound derived from the local conditions, rather than a single value based on mean temperature from a remote weather station.

## Methods

### Field sites and sampling rationale

The study was conducted in the catchment areas of two malaria clinics. Both George Town (13.0939˚N, 80.2839˚E) and Besant Nagar (13.0002˚N, 80.2668˚E) clinics consistently report positive cases of both *Plasmodium vivax* and *Plasmodium falciparum* malaria that are transmitted by *An. stephensi*. Malaria cases reported at Besant Nagar clinic from 2006 to 2011 were higher compared to that of George Town. In Besant Nagar, the Slide Positivity Rate (SPR) varied from 22.6 to 42.6, with Pf% of 2.6 to 13.7%. The corresponding values reported at George Town ranged from 9.59 to 15.8 and 0.32 to 0.9%, respectively (Source: Malaria clinic data, Corporation of Chennai, India).

The current investigation was designed as a preliminary study to develop appropriate protocols and methods for a proposed year-round longitudinal survey combining environmental monitoring along with adult and larval sampling within these transmission environments. This pilot study was conducted from late January to mid April of 2012, which represents a relatively low transmission period after the end of the northeast monsoon season of October to December 2011.

### Placement and download of temperature data loggers

Forty-two data loggers were placed across the transmission sites. The majority of the loggers were small ‘USB-mode’ loggers set to record temperature every 30 minutes (USB-Lite, Dwyer Instruments, Michigan City, IN, USA). These loggers were selected because they were relatively inexpensive and there was no prior knowledge of how many loggers might be lost during the study. The USB loggers were supplemented with a few more expensive Hobo loggers (HOBO U12-011, Onset, Cape Cod, MA, USA) to compare reliability.

Temperatures were recorded from both indoor and outdoor sites; different structure types and homes constructed using a range of materials (Table 1). Indoor structures included positions within tile-roofed, asbestos-roofed, concrete-roofed, and thatched-roofed homes. Outdoor loggers were placed in vegetation, wells, and overhead tanks. Three loggers were placed in ‘other outdoor’ locations; one in a crevice of an outdoor brick wall, one under the thatched roof of an outdoor porch and another hanging in an outdoor terrace. Within the habitats, loggers were placed in areas typically utilized by mosquitoes (e.g. in dark corners, behind furniture, within shaded vegetation, hanging inside a well etc.) rather than exposed microenvironments where it would be unlikely to find a resting adult. The position of each logger was recorded using a GPS and details of the location and structure/habitat recorded. Loggers were downloaded every two weeks. Loggers recorded from early January to April 2012 with a peak coverage period of February 6, 2012 through April 16, 2012. Due to logger malfunction or loss, the sample size varies slightly for each month (Table 1). Additionally, hourly weather station data for the city of Chennai (actually at the airport) were downloaded from the National Oceanic and Atmospheric Administration (NOAA), National Climatic Data Center [29]. The Besant Nagar clinic is located approximately 15 km west of the local weather station at Meenambakam Airport. The George Town field site is approximately 20 km northwest of this weather station

**Table 1:** Mean, minimum and maximum temperature, and mean DTR

**Month**	**Habitat/Area**	**Structure type**	**n**	**Mean temperature**	**Min**	**Max**	**Mean DTR**
				**(°C ± SE)**	**(°C)**	**(°C)**	**(°C ± SE)**
**February**	**Indoor**	Asbestos Roof	5	30.20 ± 0.02	25	36	4.39 ± 0.15
		Concrete Roof	9	29.60 ± .02	24	37	2.15 ± 0.18
		Thatch Roof	3	28.80 ± 0.08	24	36	5.78 ± 0.22
		Tile Roof	8	29.92 ± 0.02	25	35	3.40 ± 0.03
	**Outdoor**	Vegetation	5	27.41 ± 0.03	21	35	6.21 ± 0.25
		Well	3	27.97 ± 0.03	22	36	3.27 ± 0.45
		Overhead Tank	1	32.88 ± 0.15	26	45	14.88 ± 0.45
		Other Outdoor	3	29.37 ± 0.04	22	36	6.10 ± 0.28
		NOAA	1	26.26 ± 0.10	20	35	9.67 ± 0.36
**March**	**Indoor**	Asbestos Roof	6	31.89 ± 0.02	28	39	3.99 ± 0.10
		Concrete Roof	7	30.84 ± 0.01	27	36	1.37 ± 0.06
		Thatch Roof	3	30.19 ± 0.04	26	38	5.13 ± 0.21
		Tile Roof	8	31.50 ± 0.02	25	40	3.83 ± 0.13
	**Outdoor**	Vegetation	5	29.79 ± 0.02	25	38	6.03 ± 0.20
		Well	3	29.31 ± 0.03	25	40	3.64 ± 0.39
		Overhead Tank	1	33.73 ± 0.10	27	43	12.03 ± 0.25
		Other Outdoor	3	31.36 ± 0.04	25	37	7.18 ± 0.33
		NOAA	1	28.94 ± 0.08	22	36	9.07 ± 0.30
**April**	**Indoor**	Asbestos Roof	6	33.01 ± 0.03	29	39	4.41 ± 0.14
		Concrete Roof	7	31.57 ± 0.01	30	35	0.94 ± 0.07
		Thatch Roof	2	30.85 ± 0.05	27	36	3.97 ± 0.21
		Tile Roof	8	32.36 ± 0.02	28	38	3.70 ± 0.16
	**Outdoor**	Vegetation	4	30.69 ± 0.04	19	35	5.19 ± 0.25
		Well	3	30.25 ± 0.03	27	37	4.00 ± 0.46
		Overhead Tank	1	34.76 ± 0.15	29	43	11.38 ± 0.59
		Other Outdoor	3	32.35 ± 0.05	27	40	6.38 ± 0.38
		NOAA	1	30.22 ± 0.12	24	37	9.10 ± 0.35

The mean, minimum, maximum, and DTR for the different categories of data logger location subdivided by month.

### Data analysis

For each logger, on each day the minimum, maximum, mean and temperature range (DTR, the difference between daily minimum and maximum) were calculated. Additionally, the hourly temperature recorded by each logger was averaged each day to give a daily mean temperature for each logger. The average temperatures reported for loggers located in different field sites (NOAA, Besant Nagar, and George Town) were compared using 95% confidence intervals. Differences between temperatures reported by data loggers located in the same structure type, but in different field sites, were determined using a paired t-test [30]. All statistical analyses were run using SPSS (Version 20.0. IBM Corporation, Armonk, NY). Mean monthly temperatures (mean of daily temperatures in a month) and mean DTR (mean of daily temperatures recorded) were also calculated for each logger type for use in modelling parasite development.

### Thermodynamic models

The effects of temperature (T) on the EIP of the parasite was examined using a published non-linear thermodynamic model for *P. falciparum*[21]. This model describes parasite development rate (PDR, the reciprocal of the EIP) and was derived by fitting a standard temperature-development function [31] to a range of published empirical and modelling data on parasite development:

$$\mathrm{PD}R_{\;\mathrm{falciparum}}\left(T\right)=0.000112\;T\left(T-15.384\right)\sqrt{\left(35-T\right)}$$

**Figure 1:**
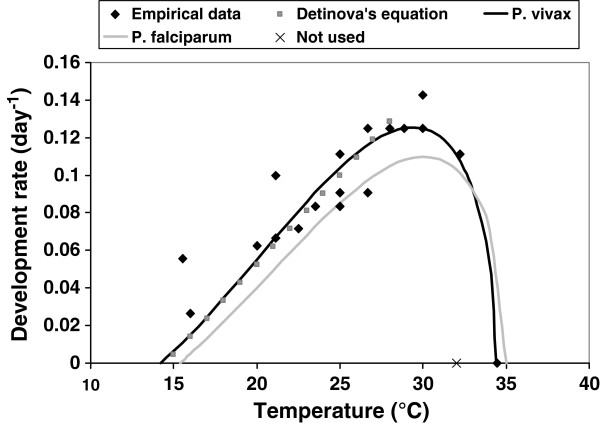
**Relationship between temperature and the development rate of *P. vivax*.** The function as proposed by Brière *et al.*[30] is fitted to a set of empirical data (see Methods for references) and the well-established Detinova equation [37] over a defined temperature range (black line). The previously published thermal performance curve for *P. falciparum* is plotted in grey. The optimal temperature for *P. falciparum* development in this curve is 30.1°C and 29.4°C for *P. vivax.*

A similar curve was generated for *P. vivax* development using a suite of empirical data [32-37] combined with estimates of development from the standard degree-day model for *P. vivax*[38] over the linear range of the Brière function (Figure 1) to give:

$$\begin{array}{c} \mathrm{PD}R_{\mathrm{vivax}}\left(T\right)=0.000126\;T\left(T-14.244\right)\\ \;\sqrt{\left(34.4-T\right)}\;\left(R^{2}=0.897\right) \end{array}$$

One empirical data point from a study in South America was omitted from the analyses, as it suggested a critical maximum temperature (CT_max_) for *P. vivax* of 32°C, yet two other studies in Indian vectors indicate parasite development above this temperature [33,34].

Mean hourly temperature and mean monthly temperature data for each data logger were used to calculate the development rates of *P. falciparum* and *P. vivax* using the thermodynamic models presented above, and subsequently converted to provide estimates of EIP. Average EIPs for each structure type were compared across each month, based on either the mean monthly temperatures (the typical way of characterizing the environment) or the mean hourly temperatures, which captures any additional effect of temperature variation that occurs through the day. The temperature development models include an upper lethal limit, set at 5°C above the CT_max_[39]. If temperatures exceed these limits the parasite is assumed to die and no estimates of EIP are possible. Curve fitting was done in SPSS (IBM SPSS v20), with the analyses and figures generated in R [40].

## Results

### Comparison between weather station data and temperatures within the urban transmission sites

**Figure 2:**
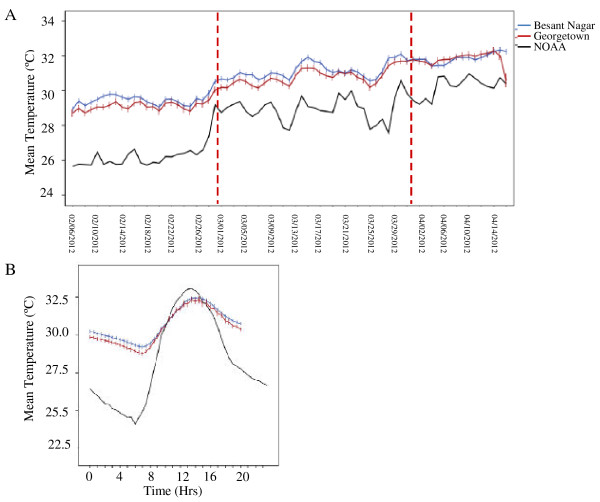
**Temperature reported by local weather station and within transmission sites. A**. Average daily temperature over sampling period in all sites. Red lines indicate the month breaks in the data. **B**. Average hourly temperature profile for loggers located at transmission sites and the nearest NOAA weather station. Within transmission sites there was less cooling during the night and this resulted in higher average temperatures. Bars represent approximate 95% confidence intervals (±2 standard errors) of the mean calculated across the hourly temperature for loggers at each site.

Environmental temperature increased over the two and a half months (February, March, first half of April) monitoring period (Figure 2A). Average daily temperatures recorded from data loggers within the local transmission sites were significantly warmer than those recorded by the NOAA weather station (Figure 2A). There were no differences between the temperatures recorded by loggers located in the same structure types between the field sites (paired t-test, t=0.92, d.f. =6, t=0.39). The lower average daily temperatures reported by the weather station were due to the fact that this location experienced cooler temperatures during the night. This effect also resulted in larger average DTRs at this location compared with the urban transmission (Figure 2B).

### Diversity of thermal environments within transmission sites

**Figure 3:**
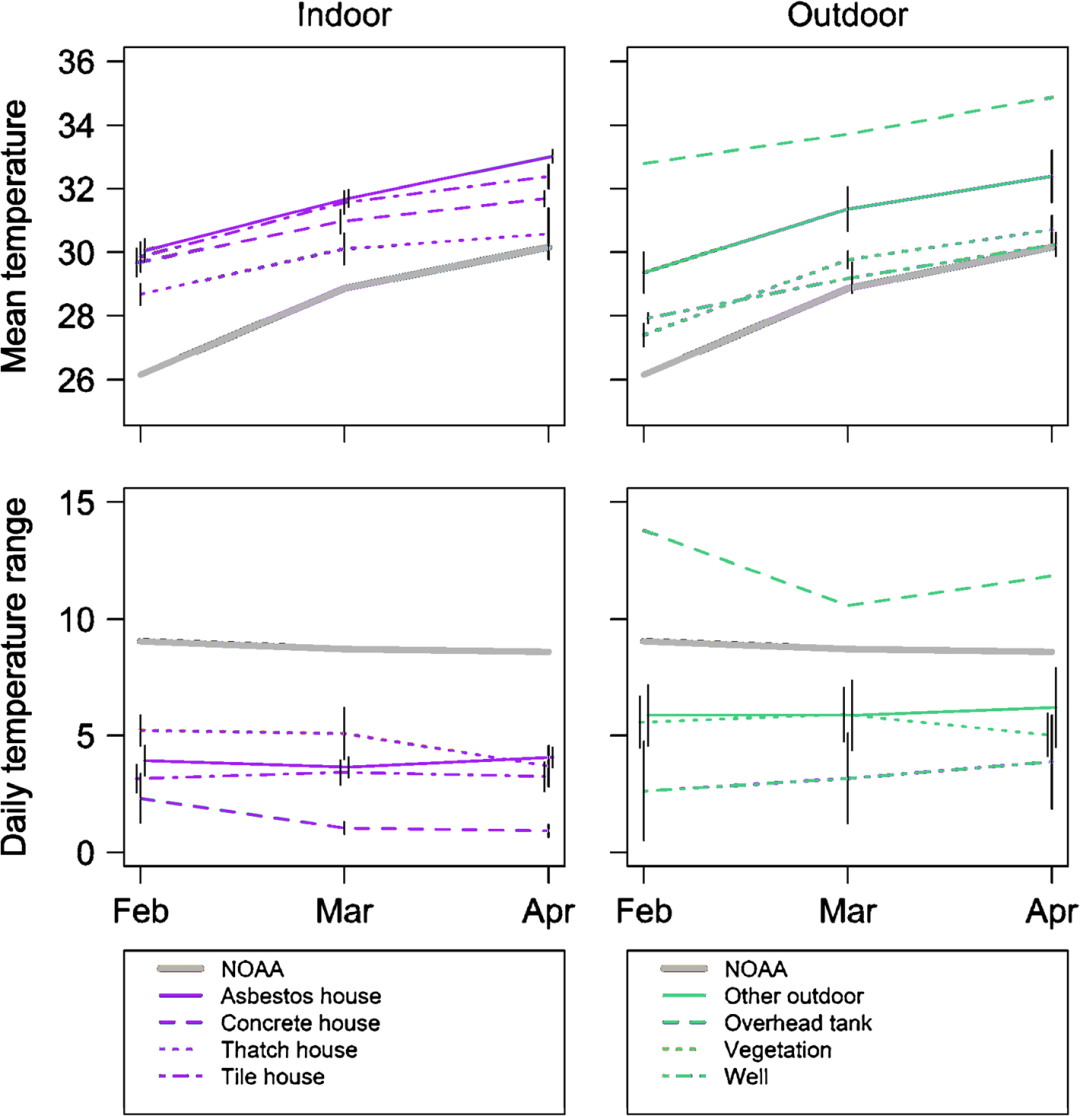
**Average temperature and DTR for different structure types (divided in indoor and outdoor structures) over a 3 month time period.** Bars represent standard errors; there are no error bars in cases in which measurements were from a single logger (NOAA and overhead tank).

Indoor environments were warmer than outdoor environments (Indoor, 95% Confidence Interval, 30.86-30.89; Outdoor, 95% Confidence Interval, 29.99-30.06) and had smaller DTRs (Indoor, 95% Confidence Interval, 3.20-3.41; Outdoor, 95% Confidence Interval, 5.92-6.43). As mean temperatures increased over the sample period, DTRs generally decreased (Figure 3). The one exception to this pattern was an overhead water storage tank located on the roof of a 5-storey apartment building that had the warmest temperatures and the largest DTRs. Among the other environments, homes with tile and asbestos roofs were the warmest, while outdoor vegetation and wells were the coolest (Table 1). Loggers placed in ‘other outdoor’ locations had the largest DTRs, and the smallest DTRs were recorded from the inside of houses with concrete roofs.

### Extrinsic incubation periods

**Figure 4:**
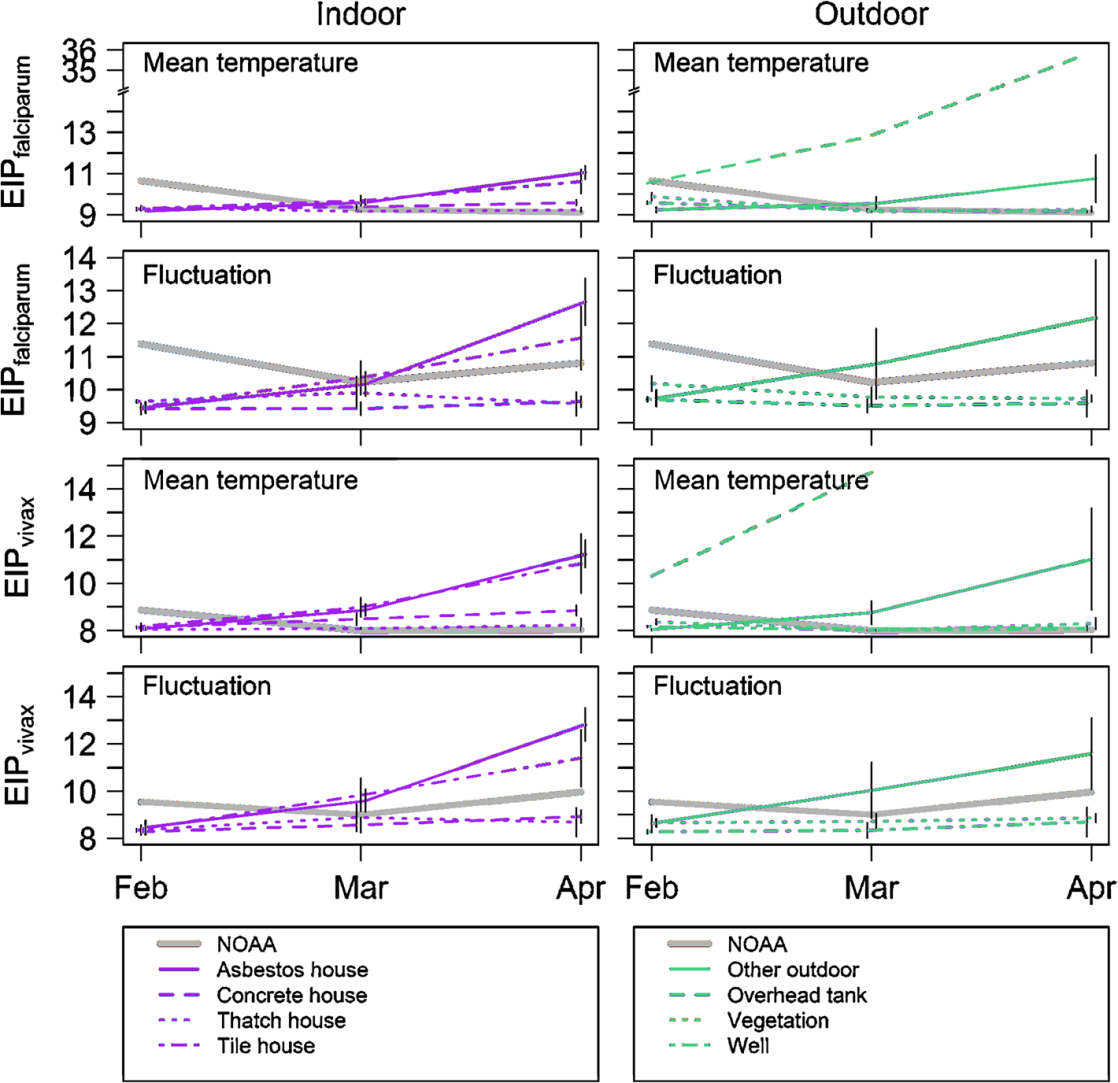
**Predicted development times of *falciparum *and *vivax *malaria (EIP in days) for different structure types (divided in indoor and outdoor structures) separated by predictions based on the mean temperature and daily variation in temperature, over approximately 3 months.** Note the increase in differences between EIP predicted from loggers and NOAA reported data when fluctuation is incorporated into EIP calculations. Bars represent standard errors.

**Figure 5:**
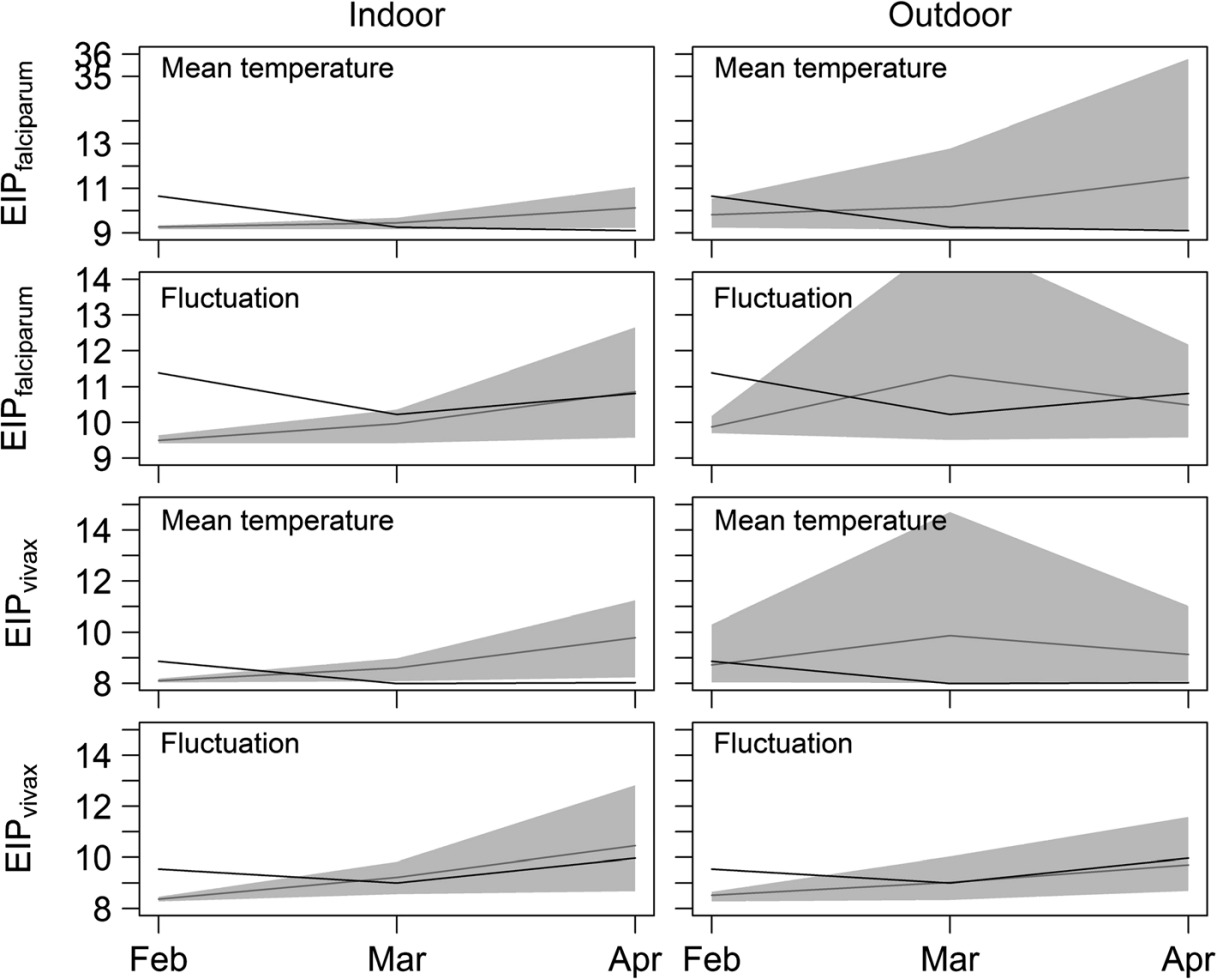
**Predicted development times of *falciparum *and *vivax *malaria (EIP in days) within the local transmission setting.** The black line indicates EIP durations as predicted using weather station data and the grey line represents the mean EIP from the ensemble of local habitat/structure types in which data loggers were placed. The grey shading indicates the potential range in EIP that exists within the transmission environment considering the diversity of microclimatic conditions extending from the coolest to warmest habitats.

With the exception of the overhead tank (Figure 4), the predicted EIPs in all indoor and outdoor structures were comparable in February (*P. vivax* 8–9 days; *P. falciparum* 9–10 days). However, all had predicted EIPs 1–2 days shorter than those predicted using the weather station data (Figure 5). As conditions warmed, predicted EIPs deviated slightly between microenvironments. The more stable conditions (< 1 day difference in predicted EIP, comparing February with April) were indoors in concrete-roofed and thatch-roofed houses, and outdoors in vegetation and wells. The houses with asbestos and tile roofs (indoor) and other outdoor habitats showed larger increases in predicted EIP over time. The shortest EIPs were predicted to be in warm stable habitats such as concrete and thatch-roofed houses and wells (Figure 4). Across the different environments, predicted EIPs ranged from 2 days shorter to 3 days longer than the EIPs based on the weather station temperatures.

Temperatures in the overhead tank were well beyond the optimum temperature for *P. falciparum* and *P. vivax* development (Figure 4). Increases in temperature over the 3 months in this structure led to substantial changes in predicted EIP for *P. falciparum* and blocked development of *P. vivax* as mean temperatures exceeded the CT_max_ (Figure 4). For most resting habitats, incorporating the effects of daily temperature fluctuations had small effects on predicted EIP, acting generally to reduce variation between microenvironments. Effects of fluctuation were slightly more marked for the predicted EIPs calculated using weather station data as the daily temperature variation reported from this location was roughly double that of most other habitat types. For these data, temperature variation acted to prolong predicted EIP in the warmest period relative to mean temperatures alone (Figure 4).

As a method to consolidate these diverse data, the estimates of the EIPs from the different microhabitat types were averaged to provide an ‘ensemble mean’ for the local transmission settings and were coupled with the upper and lower bounds of possible EIPs based on the existing environmental extremes (Figure 5). This method of presenting an ensemble mean together with an estimate of the range follows the approach commonly used to synthesize outputs from multiple Global Climate Models. In most instances, the EIP calculated from the ensemble mean differed from the estimates of EIP based on weather station data (differences in absolute value and also trends across time). More striking is the extent of the environmental envelope representing potential EIPs within the transmission setting. These extremes of potential EIP sometimes deviate from the weather station estimates by >20 days (note that in some cases predicted range of potential EIP declines as certain micro-environments become too hot to support successful parasite development).

## Discussion

The primary aim of the current study was to determine methods for sampling temperatures within transmission environments and to propose these as a framework for better understanding local transmission ecology. The empirical data presented serve as a pilot study for a more extensive longitudinal monitoring programme and so are relatively limited in scope (i.e. they are not themselves intended to provide an exhaustive evaluation of the transmission environment in the urban slums in Chennai). Nonetheless, the study illustrates the benefits of examining the variation in temperature between different potential mosquito resting habitats and potential implications for malaria transmission.

Most studies that consider the role of temperature in malaria transmission use temperatures reported by local weather stations. The current study revealed that the temperatures within the local transmission sites were warmer and more varied than those recorded by the weather station at the airport. The temperature loggers were located in a densely packed urban environment that showed less cooling at night (Figure 2B). Such ‘urban heat islands’ have been reported elsewhere in the literature [41,42] and tend to experience higher average daily temperatures than surrounding areas via effects on night-time temperatures.

Within the transmission sites, indoor temperatures remained warmer and were more stable than those recorded for outdoor environments. This type of thermal buffering has been reported in other studies, both in general terms [43] and with specific reference to malaria transmission [3,44]. With the exception of the overhead tank, which proved to be something of an extreme environment, differences in mean temperatures between microhabitats were relatively small. Even so, variation between environments led to differences in the predicted EIPs of 1–4 days for both *P. falciparum* and *P. vivax*. Adding the effects of daily temperature variation had relatively little effect because, in contrast to other parts of the world [17], the DTRs themselves were relatively small and consistent across habitats.

*Anopheles stephensi* exhibits both endophilic (indoor resting) and exophilic (outdoor resting) behaviour. The current study took a broad snap shot of temperatures available for adult mosquitoes, but it is possible that *An. stephensi* only utilizes a subset of these environments. Further, temperatures will likely vary within structure types (i.e. at different positions within a single house type) and so the temperatures experienced by mosquitoes could depend on the subtleties of the precise resting position. There is some indication that adult anopheline mosquitoes can avoid the warmest locations [45], but there is little evidence for precise behavioural thermoregulation [46]. Additionally, it has been shown that expression of mosquito heat-shock proteins increase in response to thermal stress [47] and these proteins have been shown to interact with *Plasmodium* development [48]. The extent to which these interactions would affect EIP in these environments is unclear, but should be considered in future studies. Further study on the activity patterns of *An. stephensi* in this urban environment and resting preferences within and between structure types is clearly required. For the current study there was no systematic mosquito sampling, although case data from the malaria clinics indicated there was active malaria transmission in these areas during the study period and immature forms of *An. stephensi* were found in the field sites, indicating that *An. stephensi* was utilizing some portion of the environments sampled. Precise behavioural data coupled with appropriate high resolution environmental data would refine understanding of the temperatures mosquitoes experience during parasite development. However, very few studies characterize mosquito resting behaviour in detail or couple entomological measures with site-specific estimates of microclimate.

Local meteorological station data provide a single measure of temperature and hence, generate a single estimate of temperature-dependent traits such as EIP. In contrast, the multiple data loggers placed within the transmission environment provide a measure of the temperature envelope in which mosquitoes live. In the current study, the EIPs based on the weather station data did not consistently align with the EIPs based on the ensemble mean temperatures derived from the different microhabitat types and clearly failed to capture the enormous amount of potential variation that exists within the transmission setting. The use of a distribution of temperatures does not make predictions of life history traits more ‘precise’, but it does make them more accurate in the sense that they represent the actual environment where mosquitoes rest and transmission occurs. If a single measure is required, the use of an ‘ensemble mean’ based on the average temperatures recorded across the local microhabitats should provide a more robust characterization of the transmission environment than the remote meteorological station data.

This study emphasizes the complexity of the thermal environment and its equally complex interaction with parasite development. Understanding transmission dynamics requires some consideration of this complexity. Even small absolute changes in EIP of 1–3 days can have marked impacts on transmission risk. The average daily probability of survival for *An. stephensi* has been estimated as 0.810 [49]. With an EIP of 10 days this means that approximately 12% of adult mosquitoes would live long enough to be able to transmit malaria. Increasing EIP to 12 days reduces this percentage to 8.5%, while reducing EIP to 8 days increases the value to about 18.5%. All else being equal, these changes would alter transmission intensity by approximately −45% to +50% [17].

Gaining knowledge of the temperatures experienced by mosquito vectors in the field is an important step towards a better understanding of temperature as a driver of malaria transmission. Data on the natural thermal environments of vectors are also valuable for contextualizing laboratory work on mosquito-parasite interactions. The majority of laboratory studies on vector competence and host-parasite interactions are conducted at a constant temperature of around 26°C. While only a limited study, the data from the urban transmission sites in Chennai suggest mosquitoes rarely encounter such temperatures and are subject to both higher mean temperatures and daily temperature fluctuations.

## Competing interests

The authors declare that they have no competing interests.

## Authors’ contributions

MBT and AE designed the experiment with inputs from MTM and AFR. LJC participated in study design and wrote the manuscript with assistance from MBT and AE. ST, SR and JAJ executed data logger placements and systematically downloaded the data in the field. KPP, ST and SR contributed to temperature data analysis. KPP applied models to temperature data collected. All authors read and approved the final manuscript.
